# Processed Pearl Millet Improves the Morphology and Gut Microbiota in Wistar Rats

**DOI:** 10.3390/foods14152752

**Published:** 2025-08-07

**Authors:** Jaqueline Maciel Vieira Theodoro, Lucimar Aguiar da Silva, Vinícius Parzanini Brilhante de São José, Nathaniel Baldwin Willis, Renata Celi Lopes Toledo, Mariana Grancieri, Carlos Wanderlei Piler Carvalho, Joseph Francis Pierre, Bárbara Pereira da Silva, Hércia Stampini Duarte Martino

**Affiliations:** 1 Department of Nutrition and Health, Federal University of Viçosa, Viçosa 36570900, Brazil; jaquemacielvieira@gmail.com (J.M.V.T.); lucimar.aguiar@ufv.br (L.A.d.S.); vinicius.sao@ufv.br (V.P.B.d.S.J.); renatacelly@yahoo.com.br (R.C.L.T.); barbara.p.silva@ufv.br (B.P.d.S.); 2 Department of Nutritional Sciences, College of Agriculture and Life Science, University of Wisconsin-Madison, Madison, WI 53706-1527, USA; nbwillis@wisc.edu (N.B.W.); jpierre@wisc.edu (J.F.P.); 3 Department of Pharmacy and Nutrition, Federal University of Espírito Santo, Alegre 29500-000, Brazil; marianagrancieri@gmail.com; 4 Embrapa Food Technology, Rio de Janeiro 23020-470, Brazil; carlos.piler@embrapa.br

**Keywords:** extrusion cooking, germination, hemoglobin depletion/repletion, intestinal health

## Abstract

This study evaluated the effect of pearl millet subjected to different processing on the intestinal health of rats. The animals were fed a standard iron-free diet (28 days) (hemoglobin depletion: 8.65 + 1.40 g/dL of hemoglobin). Subsequently, they were divided into four groups for hemoglobin repletion (21 days): standard diet + ferrous sulfate (SD + FS); standard diet + non-germinated open-pan cooked millet flour (SD + NGOPCMF); standard diet + germinated open-pan cooked millet flour (SD + GOPCMF); and standard diet + extrusion-cooked millet flour (SD + ECMF). Hemoglobin level did not differ among groups. The SD + NGOPCMF, SD + GOPCMF and SD + ECMF groups demonstrated a higher Chao index in the microbiome and a higher number and area of goblet cells and longitudinal muscle layer width. The SD + NGOPCMF and SD + GOPCMF groups demonstrated increased cecum weight, crypt depth, crypt thickness, and circular muscle layer width; lower fecal pH; and a higher relative abundance of Bacteroidota, while the SD + FS group showed the highest abundance of Actinobacteriota. The SD + GOPCMF group stood out for showing the lowest fecal pH, better α-diversity (Chao and Shannon index), and the highest width of the longitudinal muscle layer. In conclusion, pearl millet subjected to different processing, mainly germination, has the potential to improve the composition of the intestinal microbiota and the intestinal morphology in rats induced to iron deficiency.

## 1. Introduction

Iron is an essential nutrient for both the host and the microbiome [1]. Iron deficiency can inhibit important metabolic processes, impair bacterial growth, and disrupt the organism’s homeostasis [1,2]. On the other hand, excessive iron levels can induce oxidative stress and promote the proliferation of pathogenic microorganisms [3]. Therefore, maintaining iron levels without toxicity is vital for optimal health.

One of the strategies to combat iron deficiency or anemia is the use of iron salts with high bioavailability. However, this approach is often associated with adverse gastrointestinal effects, including constipation, gastric irritation, nausea, and metallic taste [4]. Moreover, the intake of iron salts may exacerbate inflammation and oxidative stress, since iron can generate free radicals that form reactive oxygen species (ROS) and also serves as an essential growth factor for potentially pathogenic bacteria, which can lead to dysbiosis and damage to intestinal health [5,6].

In developing countries, access to animal-derived products (meat, fish and eggs) is limited, making cereals and legumes the primary dietary sources of iron. Therefore, diversifying staple foods by incorporating crops that are natural sources of iron, such as millet, is essential, in addition to being an option for vegans, vegetarians and flexitarians [7,8]. Millet ranks sixth among the most produced crops in the world, with an area of 30 million hectares in the arid and semiarid tropical regions of Asia and Africa [9]. Although it is a good source of iron, the availability of this mineral in millet is influenced by the presence of non-heme iron, which is less easily absorbed compared to the heme iron found in animal products [10]. On the other hand, in addition to minerals, especially calcium (300 mg/kg), zinc (43 mg/kg) and iron (18 mg/kg), pearl millet (*Pennisetum glaucum* (L.) R. Br.) is a valuable source of protein (8 to 11%), dietary fiber (7 to 12%), resistant starch (average content of 3%), and other bioactive compounds, such as polyphenols (Diosmin and Cyanidin) and fatty acids (mainly polyunsaturated), which confer several health benefits, including promoting intestinal health, which can favor nutrient absorption [11,12].

The inclusion of pearl millet as a strategy to mitigate metabolic changes that are harmful to the body’s homeostasis has been investigated, and research has demonstrated its potential antioxidant and anti-inflammatory properties, as well as its benefits for intestinal health in animals [13,14,15], in addition to the glucose-lowering effect in individuals with impaired glucose tolerance [16]. Furthermore, studies have also demonstrated that certain processing techniques, such as germination and extrusion cooking, can enhance the nutritional quality of this cereal [12,17,18] and its effect on glycemic and insulinemic control, food intake and appetite sensation in eutrophic adults [19]. However, pearl millet processed by germination and extrusion acting on intestinal health has not yet been evaluated. Therefore, the present study aimed to evaluate the effect of pearl millet subjected to different processing methods on the intestinal health of iron-deficient rats, using the hemoglobin depletion/repletion model. The hypothesis is that non-germinated, germinated and extruded pre-cooked millet flours, as a source of iron and bioactive compounds, would improve the composition of the intestinal microbiota and the morphology of the intestine in rats induced to iron deficiency.

## 2. Materials and Methods

### 2.1. Processing of Millet Grains and Flour Preparation

Pearl millet grains (*Pennisetum glaucum* (L.) R. Br.), hybrid ADRg 9070, kindly donated by Atto Sementes (Rondonopolis, Brazil), harvested during the second growing season of 2021 in Itiquira, Mato Grosso, Brazil, were used.

The grains were germinated under controlled conditions of temperature (30° ± 2 °C) and humidity (90%) for 24 h (National Mfg. Co., Lincoln, NE, USA) and subsequently dried at 50 °C for 4 h (Macanuda Hauber, Joinville, Brazil) [12]. The extrusion process was performed in an Evolum HT25 (Clextral Inc., Firminy, France) twin-screw co-rotating extruder with a screw diameter of 25 mm and a length-to-diameter ratio of 40:1. The following temperature profile of the ten heating zones was used, from feeding to the output: 25, 40, 50, 90, 100, 110, 110, 110, 120, 120 °C and feed rate and moisture were kept constant at 14% (injection water pump) and 10 kg/h (gravimetric feeder), respectively. The puffed extrudates were then dried at 60 °C for 2 h in a 400/6ND ventilated oven (Nova Ética^®^, São Paulo, Brazil) [12].

The heat treatment of the ungerminated and germinated millet grains took place in a pan (180 °C/40 min) and then they were dried (50 °C/24 h) (Nova Ética^®^, São Paulo, Brazil) [11,12]. To produce the flours, the pre-cooked whole grains were ground in a mill equipped with a 1 mm sieve (TREU, Rio de Janeiro, Brazil), operating at a capacity of 500 kg/h. The flours were then stored in a sealed plastic bag at −20 °C ± 1 °C until analysis.

The flours were designated as follows: non-germinated open-pan cooked millet flour (NGOPCMF: 68.24% carbohydrates, 11.16% protein, 11.88% total dietary fiber, 10.45% insoluble fiber, 1.43% soluble fiber, 3.38 g/100 g resistant starch, 5.58% lipids, and 4.49 mg/100 g iron), germinated open-pan cooked millet flour (GOPCMF: 67.68% carbohydrates, 11.46% protein, 11.91% total dietary fiber, 11.43% insoluble fiber, 0.49% soluble fiber, 3.53 g/100 g resistant starch, 5.03% lipids, and 4.18 mg/100 g iron) and extrusion-cooked millet flour (ECMF: 74.87% carbohydrates, 10.97% protein, 7.96% total dietary fiber, 6.82% insoluble fiber. 1.14% soluble fiber, 0.32 g/100 g resistant starch, 2.82% lipids and 5.64 mg/100 g iron).

### 2.2. Experimental Design

Thirty-two male *Wistar* rats (*Rattus norvegicus*), 21 days old, were distributed in individual stainless-steel cages with controlled temperature conditions (22 ± 2 °C) and a 12 h photoperiod. The rats were induced to iron deficiency through the hemoglobin depletion/repletion method. The animals were fed an AIN-93G standard diet (SD) [20], containing an iron-free mineral mix, to deplete hemoglobin levels and induce iron deficiency (28 days). After this phase, the hemoglobin concentration was evaluated by the colorimetric assay kit (Labtest^®^, Lagoa Santa, MG, Brazil). Subsequently, during the repletion phase, the animals were randomly assigned to four groups for treatment groups, ensuring homogeneity based on mean hemoglobin levels (8.65 ± 1.40 g/dL) and body weight (263.53 ± 23.05 g). During treatment, iron was provided in the form of ferrous sulfate or available on millet flours, at a standardized concentration of 13.29 ppm per treatment [21].

Four experimental groups were used (n = 8/group): standard diet with ferrous sulfate (SD + FS), standard diet with non-germinated millet flour cooked in an open pan (SD + NGOPCMF), standard diet with germinated millet flour cooked in an open pan (SD + GOPCMF), and standard diet with millet flour processed by extrusion (SD + ECMF). Animals received deionized water ad libitum and a controlled diet, which was weighed daily over a 21-day period. Weight gain and hemoglobin levels were monitored weekly. On day 50, the animals were anesthetized with isoflurane (Isoforine, Cristália^®^) and euthanized via cardiac puncture. The proximal colon was collected, washed with phosphate-buffered solution (PBS), and kept in 10% formaldehyde at room temperature. The cecum was weighed, and its contents were collected and immediately stored at −80 °C.

The study was carried out in accordance with the ethical principles for animal experimentation and was approved by the Animal Use Ethics Committee of the Federal University of Viçosa (CEUA/UFV; process no. 05/2022).

### 2.3. Hemoglobin Measurement

Hemoglobin levels were determined using a Labtest colorimetric kit (Ref 43—hemoglobin, and Ref 47—standard hemoglobin) (Labtest^®^, Lagoa Santa, MG, Brazil), using the absorbance reading of a standard hemoglobin solution as a reference. The reading was performed on a Multiskan™ GO spectrophotometer (Thermo Fisher Scientifics; Waltham, MA, USA), at a wavelength of 540 nm.

### 2.4. Bristol Scale and Feces Color

The Bristol Stool Form Scale (BSFS) was used to obtain information about intestinal transit and bowel function, as described by Martinez and Azevedo [22]. Analysis of feces color was performed by macroscopic observation, adapting the classification proposed by Silveira Júnio [23].

### 2.5. Fecal pH

A 0.4 g aliquot of cecum content and 4 mL of distilled water were mixed using a vortex for 20 s. The pH was then measured by inserting a glass electrode (Bel Engineering^®^) directly into the suspension [24].

### 2.6. DNA Extraction, Sequencing and Data Analysis

Genomic DNA was extracted and purified from cecal content samples using the QIAamp^®^ DNA Mini Kit (Qiagen, Hilden, Germany), following the manufacturer’s protocol.

Extracted DNA samples were submitted to NovoGene Co., Ltd. (Beijing, China) for amplification and paired-end sequencing of the V3-V4 region of the 16S rRNA gene (forward: 341 F: 50-CCTACG-GGNGGCWGCAG-30 and reverse: 805 R: 50-GACTACHVGG-GTATCTAATCC-30) on an Illumina MiSeq platform (Illumina Inc., San Diego, CA, USA). The resulting libraries were demultiplexed and paired in QIIME2 (version 2023.7) [25]. Paired-end reads were passed to DADA2 for trimming, dereplication, denoising, and filtering [26]. The quality threshold was set at 30, and chimeric sequences were removed. A total of 1,654,918 reads were generated with a median of 51,716 reads per sample, while 174 sequences were detected. After removing singletons and sequences present in fewer than 10% of the sample, 117 unique sequences remained. Taxonomy was assigned with a naïve-Bayes classifier, trained with the 341F/805R amplicons against the Silva 138.1 nr99 SSU database [27].

Microbiome analysis and visualization were performed using MicrobiomeAnalyst (Xia Lab, McGill University, Montréal, QC, Canada) [28]. Alpha diversity was assessed using the Chao 1, Shannon, and Simpson indices, while β diversity among groups was evaluated based on the Bray–Curtis dissimilarity index and tested using PERMANOVA. The linear discriminant analysis effect size (LEfSe) method was used to investigate differences in gut microbiota and identify significant bacterial biomarkers among groups [29].

### 2.7. Short-Chain Fatty Acids (SCFA) Concentration

The concentration of short-chain fatty acids (SCFAs) in the cecum content was analyzed using the methodology proposed by [30]. SCFA quantification was determined by high-performance liquid chromatography (HPLC), using a Dionex Ultimate 3000 Dual Detector HPLC system (Dionex Corporation, Sunnyvale, CA, USA), as described by [31]. Acetic, propionic, butyric and valeric acids were used as standards for the calibration curve.

### 2.8. Histomorphometry Analysis of the Colon

Histological sections (3 μm thick) of the proximal colon were stained with hematoxylin and eosin using a rotary microtome (Reichert-Jung^®^, Genossen, Germany) and examined under an Olympus BX43 optical microscope with a 4x objective [31]. Crypt depth, crypt thickness, circular and longitudinal muscle layer thickness, and the number and area of goblet cells were measured in twenty fields per animal, using Image-Pro-Plus^®^ software, version 4.5 (Media Cybernetics, Rockville, MD, USA).

### 2.9. Correlation Analysis of Gut Microbiota and SCFA Concentration with Markers of Inflammation and Oxidative Stress

To assess the relationship between changes in the relative abundance of intestinal microbiota and changes in markers of inflammation and oxidative stress, a Pearson correlation analysis was performed. The relative abundance data were log-transformed to account for compositionality of ASV variables prior to running your correlations. Markers of inflammation (tumor necrosis factor gene expression (mRNA TNF)) and oxidative stress (nuclear factor erythroid 2 gene expression (mRNA NRF2); malondialdehyde concentration (MDA); nitric oxide concentration (NO); superoxide dismutase activity (SOD); and catalase activity (CAT)) had been previously evaluated and published [32]. The expression of TNF and NRF2 mRNA levels in the liver was analyzed by real-time polymerase chain reaction (RT-qPCR), and quantification was performed on the StepOnePlus™ Real-Time PCR System, using the SYBR-Green fluorescence quantification system and Primer Express software system, v3.0.1 (Applied Biosystems, Foster City, CA, USA). The concentration of MDA and NO and the activity of the antioxidant enzymes SOD and CAT were evaluated in the liver tissue homogenate, and the reading was performed on a Multiskan™ GO spectrophotometer (Thermo Fisher Scientific; Waltham, MA, USA).

### 2.10. In Silico Docking

Interactions of the phenolic compounds previously identified in millet flours (Diosmin and Cyanidin 3-O-rutinoside betaine) [12] with the markers of inflammation and antioxidant potential (Nrf2 gene expression, SOD, TNF-alpha gene expression and catalase activity) were analyzed by in silico analysis. The 3D crystal structures were obtained from the Protein Data Bank (PDB) website (http://www.rcsb.org/pdb/home/home.do): Nrf2 (PDB: 4xmb), SOD (PDB: 1pu0), TNF-alpha (PDB: 2az5), and catalase (PDB: 1dgf). Diosmin and Cyanidin 3-O-rutinoside betaine were used as ligands, and their structures were recovered from the PubChem Compound database (https://pubchem.ncbi.nlm.nih.gov/, accessed on 1 April 2025).

Flexible torsions, charges, and grid size were carried out by AutoDock Tools, and docking calculations were performed using AutoDock Vina [33]. The binding pose with the lowest binding energy (highest binding affinity) was selected as a representative image to be visualized in the Discovery Studio 2016 Client (Dassault Systemes Biovia Corp^®^, San Diego, CA, USA).

### 2.11. Statistical Analysis

The experiment was performed with a sample size of 8 animals per group, with each animal representing a repetition. The results were assessed for normality using the Shapiro–Wilk and Kolmogorov–Smirnov tests. Subsequently, the data were subjected to one-way ANOVA and the Newman–Keuls post hoc test. Differences in beta diversity were analyzed using pairwise PERMANOVA. In the taxonomic analysis, comparisons of relative abundance between groups were made at the phylum level by one-way ANOVA and the Newman–Keuls test. Pearson’s correlation analysis was used to assess the relationship between comparative alterations in intestinal microbiota relative abundance and markers of inflammation and oxidative stress. The ASV variables were previously transformed into log ratios to take into account their compositional nature. All statistical analyses were performed using GraphPad Prism software (GraphPad Software, San Diego, CA, USA), version 9.0. The significance level of 5% (*p* < 0.05) was established for all tests.

## 3. Results

### 3.1. Effect of Processed Pearl Millet on Food Intake, Body Weight, Hemoglobin Levels, Cecum Weight and Intestinal Transit in Rats

**Figure 1:**
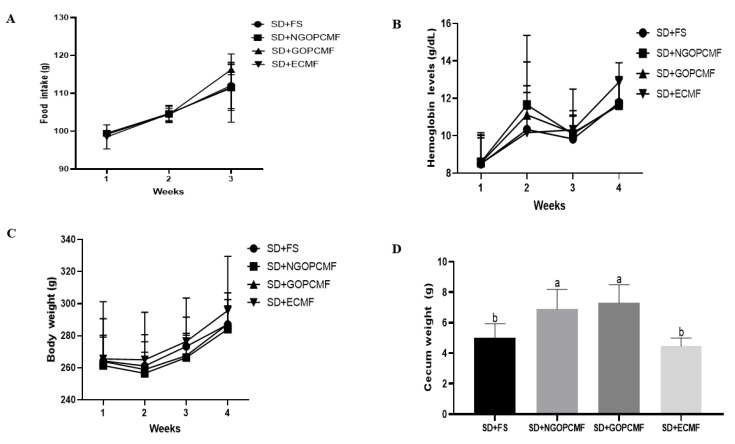
Effect of processed pearl millet on food intake (**A**), hemoglobin levels (**B**), body weight (**C**) and cecum weight (**D**) after three weeks of treatment. Means without letters indicate no difference, and different letters indicate significance by the Newman–Keuls test, at 5% probability (n = 8/group). SD + FS: standard diet + ferrous sulfate; SD + NGOPCMF: standard diet + non-germinated open-pan cooked millet flour; SD + GOPCMF: standard diet + germinated open-pan cooked millet flour; SD + ECMF: standard diet + extrusion-cooked millet flour.

Throughout the three weeks of the repletion period, no differences (*p* > 0.05) were observed in food intake, hemoglobin levels, or body weight among the experimental groups (Figure 1A–C). However, at the end of the experiment, the SD + NGOPCMF and SD + GOPCMF groups showed higher cecum weight (*p* < 0.05) compared to other groups (SD + ECMF and SD + FS) (Figure 1D).

According to the Bristol scale, the feces of the SD + NGOPCMF and SD + GOPCMF groups were darker in color in relation to the other groups (SD + ECMF and SD + FS) (Table 1). In terms of consistency, the groups treated with pre-cooked millet flour presented smoother and softer feces compared to the group treated with ferrous sulfate. Nevertheless, all groups were classified as having a normal intestinal transit pattern (Table 1).

### 3.2. Effect of Processed Pearl Millet on Fecal pH and Short-Chain Fatty Acids Concentration

The SD + GOPCMF group showed the lowest fecal pH (*p* < 0.05), followed by the SD + NGOPCMF group. No difference (*p* > 0.05) was observed between the SD + ECMF and control (SD + FS) groups. Regarding short-chain fatty acid (SCFA) concentration, no differences (*p* > 0.05) were observed among the experimental groups after the three-week treatment period (Table 2).

### 3.3. Effects of Processed Pearl Millet on Microbial Community Diversity

**Figure 2:**
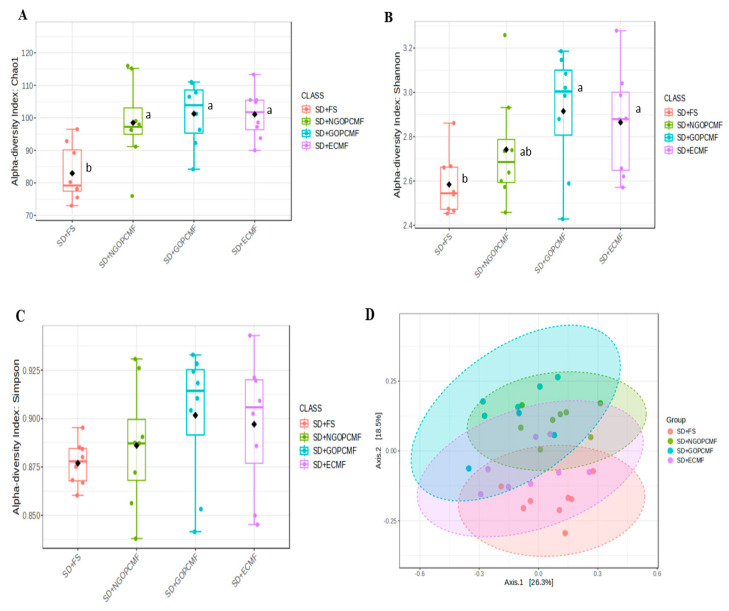
Microbial diversity of the cecal microbiome of different experimental groups after three weeks of the treatment. The α-diversity by: (**A**) Chao Index; (**B**) Shannon Index; (**C**) Simpson Index. ANOVA *p* < 0.05. The β-diversity is shown through the Bray–Curtis dissimilarity distances separated by two principal components (PCoA) (**D**). PCoA analyzed by the PERMANOVA test, *p* < 0.001. Each dot refers to one animal (n = 8/group). SD + FS: standard diet + ferrous sulfate; SD + NGOPCMF: standard diet + non-germinated open-pan cooked millet flour; SD + GOPCMF: standard diet + germinated open-pan cooked millet flour; SD + ECMF: standard diet + extrusion-cooked millet flour. Different letters indicate significance by the Newman–Keuls test, at 5% probability.

In the α diversity analysis, the SD + NGOPCMF, SD + GOPCMF and SD + ECMF groups demonstrated a higher (*p* < 0.05) Chao index compared to the control group (SD + FS) (Figure 2A). The SD + GOPCMF and SD + ECMF groups demonstrated a higher Shannon index (*p* < 0.05), while SD + NGOPCMF did not differ (*p* > 0.05) from the other groups fed with millet (SD + GOPCMF and SD + ECMF), but also did not differ (*p* > 0.05) from the SD + FS group (Figure 2B). The Simpson index did not differ (*p* > 0.05) among the groups (Figure 2C). In β-diversity, the PERMANOVA analysis demonstrated that the clustering differed among the experimental groups (*p* < 0.001), except for the SD + ECMF and SD + FS groups that did not differ from each other (Figure 2D).

**Figure 3:**
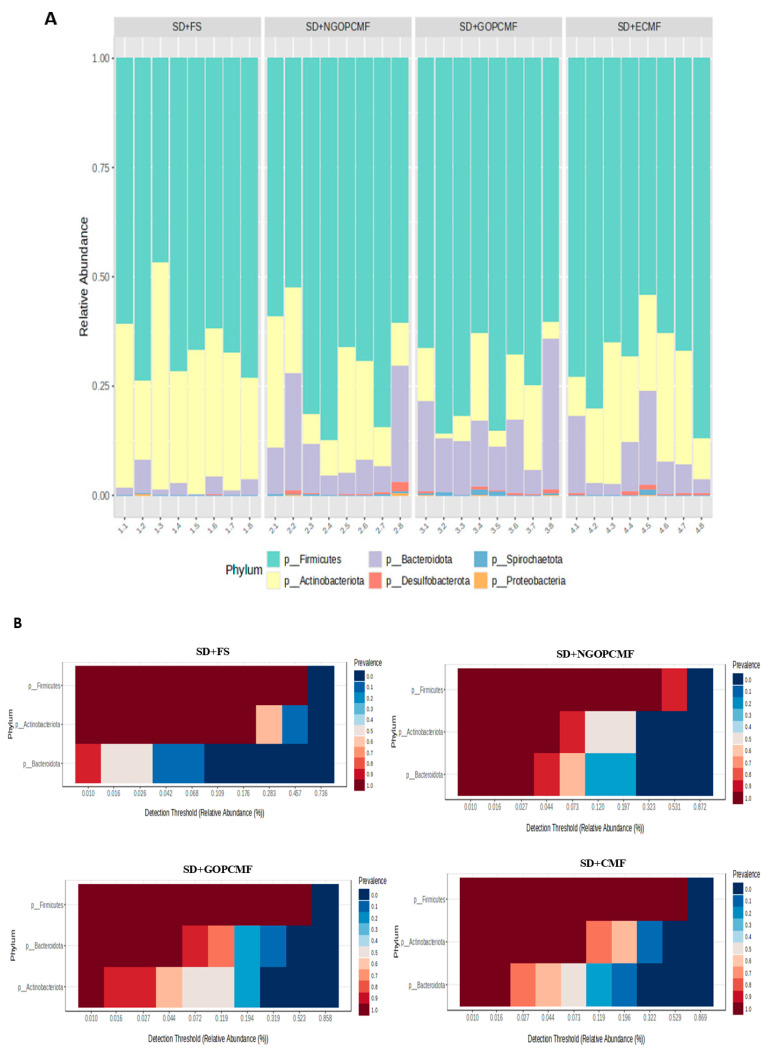
Changes in the composition of the intestinal microbiota after three weeks of treatment. Relative abundance (**A**) and core microbiome (**B**) at the phylum level (n = 8/group). SD + FS: standard diet + ferrous sulfate; SD + NGOPCMF: standard diet + non-germinated open-pan cooked millet flour; SD + GOPCMF: standard diet + germinated open-pan cooked millet flour; SD + ECMF: standard diet + extrusion-cooked millet flour. Taxa with counts < 10 were merged.

In the taxonomic classification, the phyla Actinobacteriota, Bacteroidota, and Firmicutes were the most predominant in all experimental groups (Figure 3A,B). The SD + NGOPCMF and SD + GOPCMF groups demonstrated higher (*p* < 0.05) relative abundance of Bacteroidota compared to the SD + FS group, while the SD + ECF group did not differ from the other groups. On the other hand, the SD + FS group showed the highest (*p* < 0.05) relative abundance of Actinobacteriota. No differences (*p* > 0.05) were observed in the relative abundance of Firmicutes among the experimental groups (Figure 3A,B).

**Figure 4:**
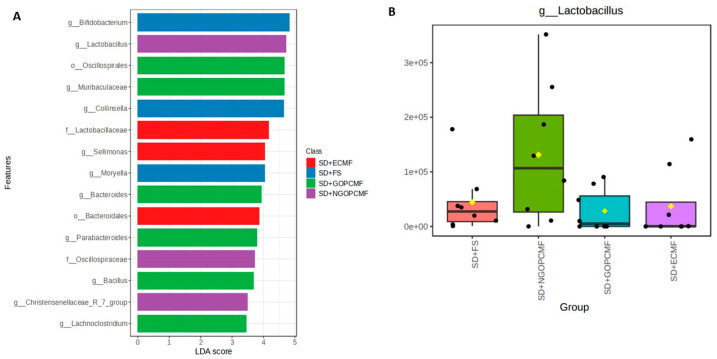

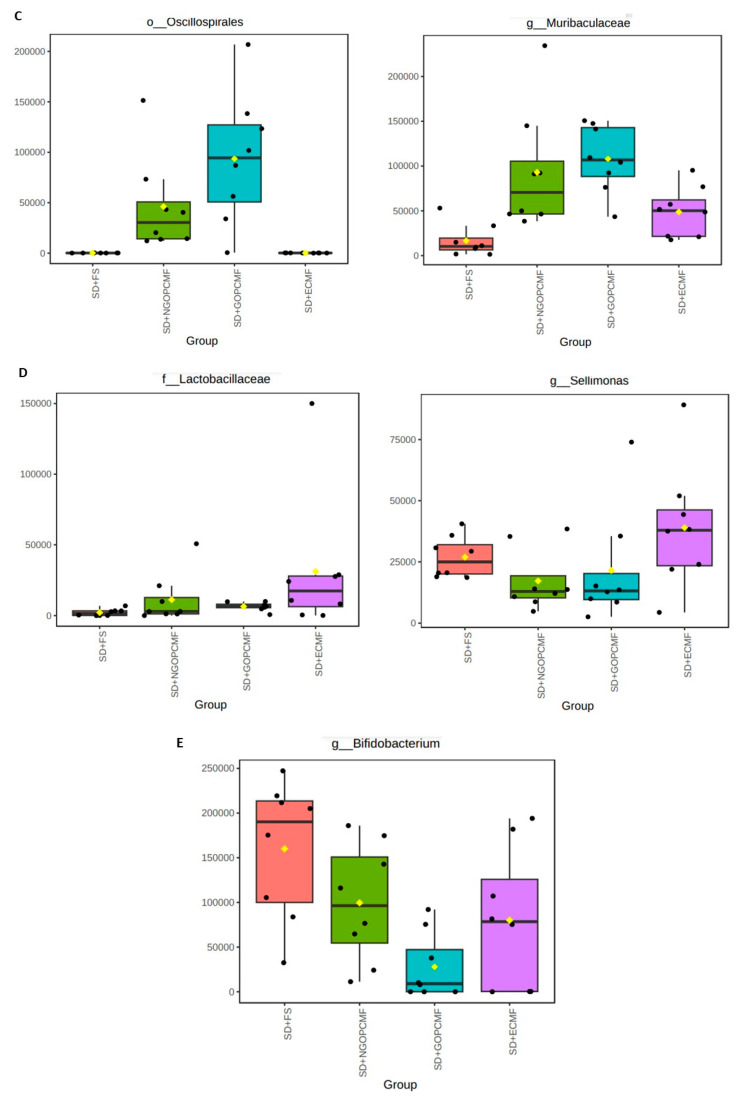
Linear discriminant analysis effect size (LEfSe) (**A**). Filtered count of significant features of relative enrichment by group: SD + NGOPCMF (**B**), SD + GOPCMF (**C**), SD + ECMF (**D**) and SD + FS (**E**). (n = 8/group). SD + FS: standard diet + ferrous sulfate; SD + NGOPCMF: standard diet + non-germinated open-pan cooked millet flour; SD + GOPCMF: standard diet + germinated open-pan cooked millet flour; SD + ECMF: standard diet + extrusion-cooked millet flour.

Regarding the LEfSe analysis, in the groups treated with millet flour, the genus *Lactobacillus*, belonging to the phylum Firmicutes, was relatively enriched (*p* < 0.05) in the SD + NGOPCMF group (Figure 4A,B). The order Oscillospirales, belonging to the phylum Firmicutes, and the family Muribaculacea, belonging to the phylum Bacteroidota, were relatively enriched (*p* < 0.05) in the SD + GOPCMF group (Figure 4A,C). The family Lactobacillaceae, followed by the genus *Sellimonas*, both belonging to the phylum Firmicutes, were relatively enriched (*p* < 0.05) in the SD + ECMF group (Figure 4A,D). In the control group (SD + FS), the genus *Bifidobacterium*, belonging to the phylum Actinobacteriota, was relatively enriched (*p* < 0.05) (Figure 4A,E).

### 3.4. Effects of Processed Pearl Millet on Intestinal Morphology

**Figure 5:**
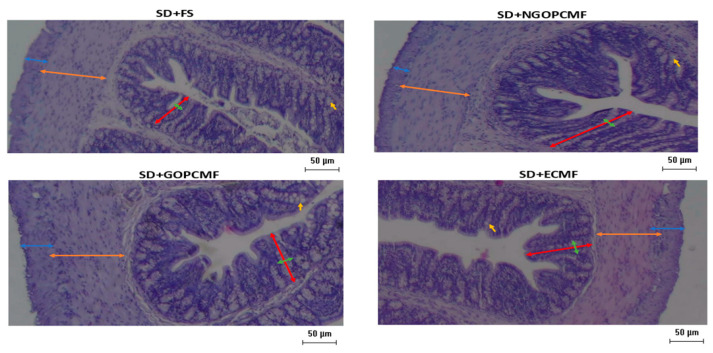
Effect of pearl millet subjected to different processing on the histomorphometry of the colon after three weeks of treatment. Yellow arrows refer to the goblet cells, red arrows refer to crypt depth, green arrows refer to crypt thickness, orange arrows refer to circular mucus layer width and blue arrows refer to longitudinal mucus layer width. SD + FS: standard diet + ferrous sulfate; SD + NGOPCMF: standard diet + non-germinated open-pan cooked millet flour; SD + GOPCMF: standard diet + germinated open-pan cooked millet flour; SD + ECMF: standard diet + extrusion cooked millet flour. Staining was carried out with hematoxylin and eosin. Bar: 50 μm. Objective magnitude: 4x.

The number and area of goblet cells were higher (*p* < 0.05) in the SD + NGOPCMF, SD + GOPCMF and SD + ECMF groups compared to the SD + FS group. Crypt depth and crypt thickness were also higher (*p* < 0.05) in the SD + NGOPCMF and SD + GOPCMF groups. For crypt thickness, the SD + ECMF group did not differ from the other millet flour groups, while the ferrous sulfate group showed lower values (Table 3 and Figure 5).

The width of the longitudinal muscle layer was higher (*p* < 0.05) in animals treated with pre-cooked millet flour (SD + NGOPCMF, SD + GOPCMF, and SD + ECMF), with the germinated flour group (SD + GOPCMF) presenting the highest values (*p* < 0.05). The width of the circular muscle layer was also higher (*p* < 0.05) in the SD + NGOPCMF and SD + GOPCMF groups in relation to the SD + ECMF and SD + FS groups (Table 3 and Figure 5).

### 3.5. Correlation of Gut Microbiota and SCFA Concentration with Inflammation and Oxidative Stress Markers

**Figure 6:**
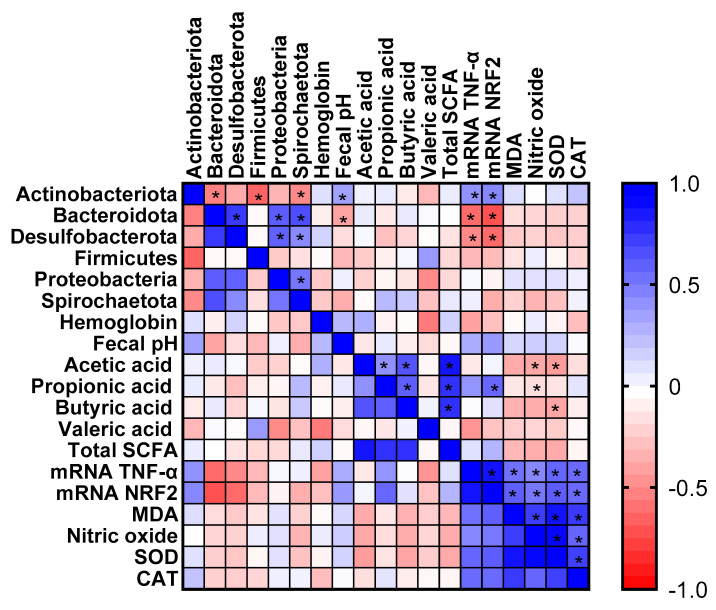
Heat map of Pearson’s correlations between alterations in intestinal microbiota relative abundance and markers of inflammation and oxidative stress after three weeks of treatment with millet subjected to different processing. Short-chain fatty acid (SCFA); tumor necrosis factor (TNF); nuclear factor erythroid 2 (Nrf2); malondialdehyde (MDA); superoxide dismutase (SOD); catalase (CAT); n = 8/group. * *p* < 0.05. Data from markers of inflammation and oxidative stress were previously published [32].

When evaluating the correlation between the comparative alterations in intestinal microbiota relative abundance with markers of inflammation and oxidative stress, the phylum Actinobacteriota was positively correlated with the gene expression of TNF (rs = 0.434; *p* < 0.05) and Nrf2 (rs = 0.474; *p* < 0.05). On the other hand, the phylum Bacteroidota was negatively correlated with the gene expression of TNF (rs = −0.588; *p* < 0.05) and Nrf2 (rs = −0.682; *p* < 0.05), as well as Desulfobacterota (TNF (rs = −0.472; *p* < 0.05) and Nrf2 (−0.588; *p* < 0.05)). Furthermore, Actinobacteriota was also positively correlated with fecal pH (rs = 0.376; *p* < 0.05) and negatively correlated with Bacteroidota (rs = −0.500; *p* < 0.05), Firmicutes (rs = −0.608; *p* < 0.05), and Spirochaetota (rs = −0.466; *p* < 0.05). The phylum Bacteroidota was positively correlated with Desulfobacterota (rs = 0.764; *p* < 0.05), Proteobacteria (rs = 0.638; *p* < 0.05) and Spirochaetota (rs = 0.688; *p* < 0.05). The phylum Desulfobacterota was positively correlated with Proteobacteria (rs = 0.619; *p* < 0.05) and Spirochaetota (rs = 0.460; *p* < 0.05). Proteobacteria was positively correlated with Spirochaetota (rs = 0.544; *p* < 0.05). In addition, markers of inflammation and oxidative stress were correlated with each other (*p* < 0.05) (Figure 6).

Regarding SCFA, propionic acid was positively correlated with Nrf2 gene expression (rs = 0.422; *p* < 0.05). Acetic acid showed negative correlations with nitric oxide (rs = −0.351; *p* < 0.05) and SOD (rs = −0.390; *p* < 0.05), while butyric acid was also negatively correlated with SOD (rs = −0.362; *p* < 0.05). Additionally, positive correlations (*p* < 0.05) were observed among the SCFAs themselves (Figure 6).

### 3.6. In Silico Docking

**Figure 7:**
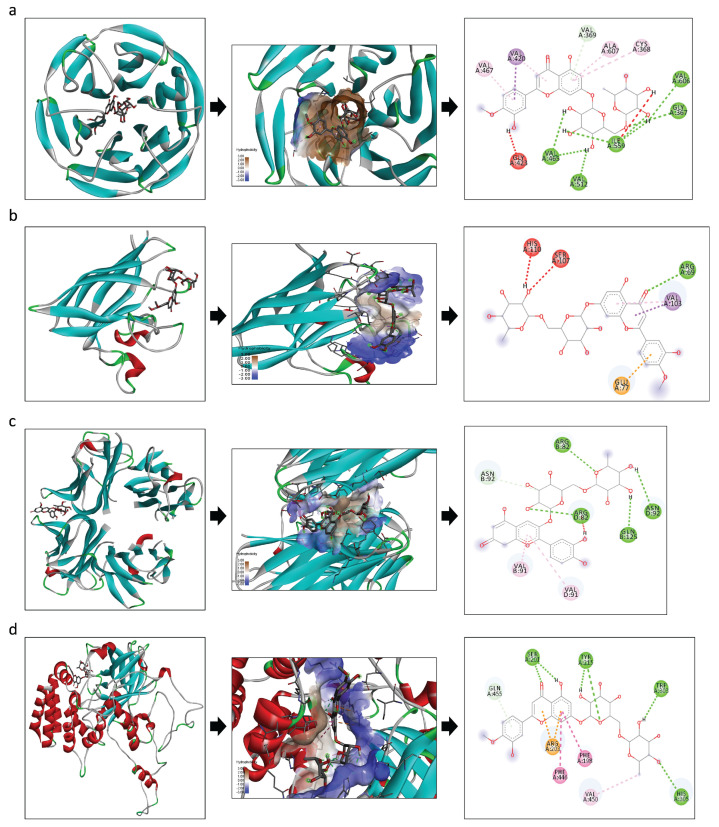
The in silico interaction of the identified phenolic compounds present in pearl millet with inflammation and oxidative markers. (**a**) Interaction between Nrf2 and Diosmin; (**b**) interaction between SOD and Diosmin; (**c**) interaction between TNF-alpha and Cyanidin 3-O-rutinoside betaine; (**d**) interaction between catalase and Diosmin. Dates analyzed by AutoDock Vina^®^ and visualized using Discovery Studio 2016 Client^®^.

Diosmin and Cyanidin 3-O-rutinoside betaine showed a good interaction with the analyzed markers Nrf2, SOD, TNF-alpha and catalase, since all of them presented low binding energy (Table 4). However, Diosmin showed the greatest interaction with Nrf2 (EFE: −11.4), SOD (EFE: −7.9), and catalase (EFE: −9.2) and Cyanidin 3-O-rutinoside betaine had the best interaction with TNF-alpha (EFE: −9.6) (Table 4 and Figure 7). Furthermore, it was observed that the main bonds between the ligands (compounds) and the markers were conventional hydrogen bonds and Pi-sigma bonds (Figure 7).

## 4. Discussion

This study evaluated the effect of pearl millet subjected to different processing methods on intestinal health in rats, using the hemoglobin depletion/repletion model, with millet flour being the only source of iron for the test groups and ferrous sulfate for the control group. The results demonstrated that pre-cooked millet flours, regardless of the processing used, have the potential to improve intestinal morphology and modulate the composition of the gut microbiota. After three weeks of treatment, rats receiving millet flours showed an enhanced Chao index, an increased number and an area of goblet cells and width of the longitudinal muscle layer. Furthermore, the SD + NGOPCMF and SD + GOPCMF groups demonstrated reduced fecal pH, different clustering in β-diversity, higher relative abundance of Bacteroidota, increased cecum weight, higher crypt depth and thickness, and wider circular muscle layer width. The SD + GOPCMF group stood out for showing the lowest fecal pH, better α-diversity (Chao and Shannon indices), and the highest width of the longitudinal muscle layer. The amount of millet flour used in this study is equivalent to approximately one teacup of flour in human dietary terms. This quantity can be easily incorporated into the daily diet through common culinary preparations such as cakes, cookies and pasta, as reported in previous studies [13].

During the treatment period, food intake, body weight and hemoglobin levels were similar among all groups, demonstrating that millet flours were as effective as ferrous sulfate in restoring iron deficiency. These findings suggest good source iron bioavailability of pearl millet flours (NGOPCMF = 4.49 mg iron/100 g flour; GOPCMF 4.18 mg iron/100 g flour; ECMF = 5.64 mg iron/100 g flour), since the hemoglobin regeneration efficiency (HRE%) was similar among the animals treated with millet flour and the control group (SD + FS = 64.8 ± 7.7^a^; SD + NGOPCMF = 67.4 ± 16.2^a^; SD + GOPCMF = 67.9 ± 13.4^a^; SD + ECMF = 77.8 ± 19.4^a^).

The high cecum weight observed in animals belonging to the SD + NGOPCMF and SD + GOPCMF groups can be due to the type of dietary fiber and the amount of resistant starch present in the non-germinated and germinated pearl millet flours. Although all experimental diets contained the same total amount of dietary fiber, predominantly insoluble fiber (provided from pearl millet flours in the test groups and cellulose in the control group), the SD + NGOPCMF and SD + GOPCMF groups also consumed soluble fiber and resistant starch inherent to the millet food matrix. These components likely contributed to the increased cecum weight. In contrast, the similarity between the SD + ECMF and SD + FS groups may be explained by the extrusion process. Resistant starch can become digestible following high-shear extrusion, thus decreasing its content and altering the dietary fiber profile [12,34]. This may have compromised the availability and physiological effect of these components in the animals’ diet.

Furthermore, the improvement in fecal consistency observed in the SD + NGOPCMF, SD + GOPCMF and SD + ECMF groups can also be attributed to the presence of soluble fiber in the pearl millet food matrix. Although no changes were observed in intestinal transit time, soluble dietary fiber contributed to the formation of smoother and softer stools, as classified by the Bristol scale. Thus, dietary fiber influenced fecal solubility, potentially enhancing nutrient absorption [35], including iron and other bioactive compounds, such as phenolic compounds, promoting improved intestinal health [36]. The darker color of the feces of the SD + NGOPCMF and SD + GOPCMF groups is probably due to the higher pigmentation of the flours compared to the SD + ECMF and SD + FS groups.

Dietary fiber and resistant starch may have localized impacts on the intestinal mucosa, stimulating the growth of epithelial cells. They may also interact with other nutrients present in the food matrix, such as proteins and fatty acids, to promote growth and improve muscle structure and function [37,38]. The higher concentration of bioactive compounds, such as polyunsaturated fatty acids, in the non-germinated and germinated pearl millet flours [12] probably facilitated nutrient interaction, leading to increased crypt depth and width of the circular muscle layer in the SD + NGOPCMF and SD + GOPCMF groups.

Furthermore, studies show that the consumption of dietary fiber, resistant starch and polyphenols is directly linked to an increase in the number and diameter of goblet cells and higher mucus production, providing higher protection for the membrane, in addition to an increase in the surface area, higher depth of the crypts and improvement in the integrity of the barrier, improving intestinal functionality and nutrient absorption [37,38,39]. These functional and morphological effects appear to occur due to increased motility of the digestive tract, leading to cell hyperplasia and/or hypertrophy [37,40], as observed in the groups treated with millet flour.

Dietary fiber and resistant starch also act as prebiotics and, upon fermentation in the intestine, promote the production of SCFA, leading to a reduction in fecal pH [35,41]. In the present study, the SD + GOPCMF group stood out for showing the highest reduction in fecal pH, followed by the SD + NGOPCMF group. Although fecal pH did not reach acidic levels, probably explaining the absence of differences in SCFA production, there were notable changes in the gut microbiota composition in the groups treated with millet flour.

The prebiotics and phenolic compounds in pre-cooked pearl millet flours improved the α and β diversity of the gut microbiota [42], promoting an increased prevalence of Bacteroidota and contributing to immune regulation by stimulating goblet cell proliferation. This likely resulted in greater mucin production, thereby strengthening the intestinal barrier, enhancing nutrient absorption, and improving overall gut health [38,43].

Increased mucin production may influence the release of growth factors and the activation of signaling pathways involved in muscle development, contributing to the increased width of the longitudinal muscle layer [37,38]. The thickened muscle layer observed in animals fed millet flour, with emphasis on the SD + GOPCMF group that showed the highest width of the longitudinal muscle layer, is associated with enhanced intestinal motility, which may facilitate more efficient transport and absorption of nutrients. This finding is consistent with a previous study, where higher expression of divalent metal transporter 1 (DMT1) was reported in animals receiving pre-cooked pearl millet flours, thus promoting better iron absorption at the enterocyte level [32].

Furthermore, the higher number and area of goblet cells, in addition to improving immune defense, possibly contributed to a higher Chao index in the animals fed millet flour in the present study. Glycine-rich mucin can be cleaved, generating energy for the growth of microorganisms, benefiting the microbiome and bacterial colonization [44].

Pearl millet presents low-specificity (soluble) fibers, easily accessible and utilized by many bacteria, and high-specificity (insoluble) fibers, accessible and fermentable by a limited number of bacteria. Thus, the dietary fiber composition of pearl millet may result in both competitive pressures for nutrient utilization, favoring the growth of diverse bacteria, and less competitive structural resources, accessible to few bacteria, favoring the growth of specific taxa [45,46]. This implies increased diversity and differential composition of the microbial community, as demonstrated in the groups treated with millet flours. The reduction in competitive pressures not only means less competition and similarity in responses between communities but also that more substrate will be available for fewer target bacteria, which can grow efficiently and significantly alter the gut microbial structure [45]. This is in line with the findings of the present study, which demonstrated an increase in the Chao index and different Bray–Curtis clustering in the groups treated with millet flours, suggesting a more diverse microbial community compared to the control group, which received only cellulose in the diet.

The SD + GOPCMF group stands out for the best α-diversity, for Chao (species richness) and Shannon (species diversity) indices, in addition to showing enrichment of Muribaculaceae, which exerts a probiotic effect and is associated with the production of SCFA via endogenous (mucin) and exogenous (dietary fiber) polysaccharides [47].

Accompanying increased microbial diversity was a higher relative abundance of Bacteroidota in the groups treated with millet flours compared to controls. On the other hand, the group treated with ferrous sulfate showed a higher relative abundance of Actinobacteriota and a reduction in Bacteroidota. The predominance of the phylum Bacteroidota, with enrichment of the genera *Lactobacillus* and *Sellimonas* and Muribaculaceae in the groups fed with millet flour, is associated with immune system regulation [47,48,49], since these may exert probiotic and anti-inflammatory effects and reduce oxidative stress by neutralizing free radicals and other reactive oxygen species [43,50]. However, the increased abundance of Actinobacteriota may lead to an exacerbated inflammatory response, since some members of this phylum are capable of producing lipopolysaccharides (LPS) and other pro-inflammatory compounds. Moreover, these bacteria can also contribute to oxidative stress by generating free radicals and other reactive oxygen species [50].

The intake of iron salts increases unabsorbed iron in the large intestine, which alters the balance between potentially beneficial and potentially pathogenic bacteria, likely leading to intestinal dysbiosis, increased inflammation, and oxidative stress [3,51]. In the present study, the Actinobacteriota phylum was positively correlated with the gene expression of TNF-alpha and Nrf2, corroborating previous findings that animals treated with ferrous sulfate demonstrated higher gene expression of these markers, along with increased levels of malondialdehyde and nitric oxide [32].

On the other hand, a negative correlation between Bacteroidota and Desulfobacterota abundance and the inflammatory and antioxidant potential markers, TNF and Nrf2, respectively, was also demonstrated. Furthermore, in silico docking analysis demonstrated interaction between the phenolic compounds (Diosmin and Cyanidin 3-O-rutinoside betaine) and markers of antioxidant potential and inflammation (Nrf2, SOD, CAT and TNF). According to the other results found, it is possible to assume that Cyanidin 3-O-rutinoside betaine acted as an anti-inflammatory compound, possibly reducing the gene expression of TNF, while Diosmin exerted an antioxidant effect, possibly reducing the gene expression of Nrf2, SOD and CAT, not requiring endogenous defense, and favoring the homeostasis of the organism. This is in line with previous studies that reported microbiota modulation and anti-inflammatory and antioxidant effects of pearl millet consumption [13,17,41].

Therefore, animals fed with pearl millet flours demonstrated increased microbial diversity and improved intestinal function, promoting the absorption of nutrients such as iron. These effects contributed not only to the recovery of iron deficiency but also supported antioxidant and anti-inflammatory responses, highlighting the potential of processed pearl millet to enhance intestinal health.

This study is innovative in investigating the effects of the processed millet on intestinal health in an iron deficiency mode. Despite the promising results, a limitation of the study should be considered. The intestinal microbiota and other variables associated with intestinal health were not evaluated in animals at the end of the depletion phase, i.e., in iron deficiency. However, these results are encouraging for replication of the study evaluating intestinal health in both the depletion and repletion phases.

## 5. Conclusions

Non-germinated, germinated and extruded pre-cooked millet flours effectively restored blood iron concentration, increased microbial diversity, and improved intestinal morphology in rats induced to iron deficiency. The SD + GOPCMF group stood out for showing the lowest fecal pH, better alpha-diversity (Chao and Shannon indices), and the highest width of the longitudinal muscle layer. These findings underscore the potential of pearl millet subjected to different processing methods, mainly germination, as a source of iron and bioactive compounds, promoting antioxidant and anti-inflammatory effects and favoring intestinal health in vivo.

## Figures and Tables

**Table 1:** Effect of processed pearl millet on Bristol scale and feces color after three weeks of treatment.

Group	Feces Color	Feces Consistency
SD + FS	Light brown-colored feces	Similarly to a sausage, but with a crack on the surface
SD + NGOPCMF	Dark brown-colored feces	Like sausage or snake, smooth and soft
SD + GOPCMF	Dark brown-colored feces	Like sausage or snake, smooth and soft
SD + ECMF	Light brown-colored feces	Like sausage or snake, smooth and soft

SD + FS: standard diet + ferrous sulfate; SD + NGOPCMF: standard diet + non-germinated open-pan cooked millet flour; SD + GOPCMF: standard diet + germinated open-pan cooked millet flour; SD + ECMF: standard diet + extrusion-cooked millet flour.

**Table 2:** Effect of processed pearl millet on fecal pH and short-chain fatty acid production after three weeks of treatment.

Variables	SD + FS	SD + NGOPCMF	SD + GOPCMF	SD + ECMF
Fecal pH	8.57 ± 0.18 ^a^	8.07 ± 0.35 ^b^	7.71 ± 0.24 ^c^	8.37 + 0.11 ^a^
Short-chain fatty acids (mM)				
Acetic acid (mM)	2.78 ± 0.50 ^a^	3.23 ± 0.84 ^a^	3.24 ± 0.69 ^a^	2.85 ± 0.43 ^a^
Propionic acid (mM)	1.69 ± 0.76 ^a^	1.73 ± 0.56 ^a^	1.37 ± 0.29 ^a^	1.12 ± 0.22 ^a^
Butyric acid (mM)	0.77 ± 0.14 ^a^	0.72 ± 0.14 ^a^	0.75 ± 0.19 ^a^	0.67 ± 0.08 ^a^
Valeric acid (mM)	0.20 ± 0.05 ^a^	0.21 ± 0.03 ^a^	0.19 ± 0.00 ^a^	0.18 ± 0.03 ^a^

Means followed by different letters in the line differ by the Newman–Keuls test at the 5% level of significance. Short-chain fatty acids were analyzed by HPLC. SD + FS: standard diet + ferrous sulfate; SD + NGOPCMF: standard diet + non-germinated open-pan cooked millet flour; SD + GOPCMF: standard diet + germinated open-pan cooked millet flour; SD + ECMF: standard diet + extrusion cooked millet flour.

**Table 3:** Effect of processed pearl millet on morphology health, after three weeks of treatment.

Variables	SD + FS	SD + NGOPCMF	SD + GOPCMF	SD + ECMF
Number of goblet cells	9.12 ± 0.86 ^b^	12.03 ± 1.47 ^a^	12.32 ± 1.62 ^a^	10.96 ± 0.74 ^a^
Goblet cell area	101.40 ± 3.64 ^c^	143.70 ± 0.86 ^a^	146.40 ± 3.87 ^a^	113.30 ± 3.65 ^b^
Crypts depth (µM)	121.40 ± 1.91 ^b^	131.10 ± 1.75 ^a^	132.80 ± 1.77 ^a^	121.50 ± 1.03 ^b^
Crypts thickness (µM)	22.54 ± 0.93 ^b^	24.67 ± 0.93 ^a^	24.52 ± 0.77 ^a^	23.56 ± 0.76 ^ab^
Longitudinal mucus layer width (µM)	38.56 ± 6.68 ^c^	50.55 ± 7.45 ^b^	73.71 ± 4.39 ^a^	48.28 ± 5.94 ^b^
Circular mucus layer width (µM)	124.10 ± 14.46 ^b^	147.40 ± 16.48 ^a^	161.30 ± 13.32 ^a^	126.10 ± 16.89 ^b^

Means followed by different letters in the line differ by the Newman–Keuls test at the 5% level of significance. SD + FS: standard diet + ferrous sulfate; SD + NGOPCMF: standard diet + non-germinated open-pan cooked millet flour; SD + GOPCMF: standard diet + germinated open-pan cooked millet flour; SD + ECMF: standard diet + extrusion-cooked millet flour.

**Table 4:** Estimated free energy binding (EFE) and chemical interactions among the phenolic compounds present in millet with the markers Nrf2, SOD, TNF-alpha, and catalase.

Compounds	Diosmin		Cyanidin 3-O-Rutinoside Betaine	
	EFE	Interacting AA Residues	EFE	Interacting AA Residues
Nrf2	**−11.4**	VAL A: 467; VAL A: 420; VAL A: 369; ALA A: 607; CYS A: 368; VAL A: 606; GLY A: 367; ILE A: 559; VAL A: 465; VAL A: 512; GLY A: 423.	−9.9	SER A: 363; SER A: 602; ARG A: 415; GLY A: 509; ALA A: 556; VAL A: 604
SOD	**−7.9**	HIS A: 110; SER A: 107; ARG A: 69; VAL A: 103; GLU A: 77	−7.4	SER A: 102; SER A: 105; GLN A: 22; SER A: 25; HIS A: 110; VAL A: 103; LEU A: 67
TNF-alpha	−7.9	LEU A: 36; TYR A: 59; TYR A: 151	**−9.6**	ARG A: 82; ASN B: 92; ASN D: 92; GLN B: 125; ARG D: 82; VAL B: 91; VAL D: 91
Catalase	**−9.2**	GLN A: 455; SER A: 201; TYR A: 215; TRP A: 303; ARG A: 203; PHE A: 446; PHE A: 198; VAL A: 450; HIS A: 305	−9.0	ARG A: 203; TYR A: 215; PRO A: 151; PHE A: 198; ASN A: 149

Docking calculations were carried out using AutoDock Vina. Negative values mean spontaneous reaction. The most potent interaction between compounds and receptor are in bold. EFE: Estimated free energy binding; AA: amino acids; Nrf2: nuclear factor erythroid 2; TNF-alpha: tumor necrosis factor-alpha; SOD: superoxide dismutase.

## Data Availability

The original contributions presented in the study are included in the article; further inquiries can be directed to the corresponding author.

## Abbreviations

The following abbreviations are used in this manuscript:

SD + FS	standard diet + ferrous sulfate
SD + NGOPCMF	standard diet + non-germinated open-pan cooked millet flour
SD + GOPCMF	standard diet + germinated open-pan cooked millet flour
SD + ECMF	standard diet + extrusion-cooked millet flour
SCFAs	short-chain fatty acids
